# High Carbon Dioxide Treatment Modulates Sugar Metabolism and Maintains the Quality of Fresh-Cut Pear Fruit

**DOI:** 10.3390/molecules25184261

**Published:** 2020-09-17

**Authors:** Di Wang, Quan Ma, Tarun Belwal, Dong Li, Wenxuan Li, Li Li, Zisheng Luo

**Affiliations:** 1 College of Biosystems Engineering and Food Science, Key Laboratory of Agro-Products Postharvest Handling of Ministry of Agriculture and Rural Affairs, Zhejiang Key Laboratory for Agri-Food Processing, National-Local Joint Engineering Laboratory of Intelligent Food Technology and Equipment, Zhejiang University, Hangzhou 310058, China; wangdi237@zju.edu.cn (D.W.); quanma@zju.edu.cn (Q.M.); tarungbpihed@gmail.com (T.B.); dong_li@zju.edu.cn (D.L.); wenxuanli@zju.edu.cn (W.L.); lili1984@zju.edu.cn (L.L.); 2 Ningbo Research Institute, Zhejiang University, Ningbo 315100, China; 3 Fuli Institute of Food Science, Hangzhou 310058, China

**Keywords:** carbon dioxide, fresh-cut fruit, pear, sugar metabolism

## Abstract

The purpose of this study is to explore the effect of 10% carbon dioxide (CO_2_) on the fruit quality and sugar metabolism of fresh-cut pear during storage. The results indicated that carbon dioxide treatment maintained fruit quality by delaying the decline of firmness and promoting the accumulation of total soluble solids (TSS). Moreover, carbon dioxide enhanced activities of sucrose synthase (SS), and sucrose phosphate synthase (SPS). The activities of amylase, acid invertase (AI), neutral invertase (NI), SS-cleavage, fructokinase (FK), hexokinase (HK), sorbitol oxidase (SOX), NAD-dependent sorbitol dehydrogenase (NAD-SDH), and NADP-SDH in CO_2_-treated fruit were inhibited. Expression levels of key genes were found to correspond with the related enzyme activities. As a result, the accumulation of glucose, fructose, sorbitol, and sucrose were accelerated by CO_2_, which were 12.58%, 13.86%, 24.7%, and 13.9% higher than those of the control at the end of storage, respectively. The results showed that CO_2_ could maintain the quality of fresh-cut pears by regulating the conversion of various sugar components to enhance soluble sugars content.

## 1. Introduction

Fresh-cut fruits and vegetables are largely consumed as daily food to meet the nutritional requirements. As a popular fruit, the consumption of fresh-cut pears is also growing rapidly. However, the cutting procedure could lead to increased respiratory, ethylene, quality loss, and subsequent changes in the carbohydrate content of fresh-cut pear fruit. Carbohydrates not only provide energy for the overall development of fruits, but they are also essential determinants of fruit quality [1]. Moreover, sugars protect cell membranes, retard electrolyte leakage, and enhances antioxidant capacity by regulating osmotic pressure [2].

Sugars are synthesized from photosynthesis of leaves and accumulate in fruits through a series of physiological steps [3]. The accumulation of soluble sugars during fruit development has a significant impact on the sweetness of fruits [4]. The sugar catabolism of postharvest fruits leads to changes in the composition and content of soluble sugars, which affect fruit flavor, a critical criterion for product acceptance by the consumer [5]. Generally, it is believed that the sugar content and composition of postharvest fruits are affected by the sucrose/hexose conversion cycle pathway [6].

In postharvest fruits, sucrose can be catalyzed and synthesized by the action of sucrose synthase (SS) and sucrose phosphate synthase (SPS), and is converted into fructose and UDP-glucose (UDPG) via the catalytic reaction of SS-cleavage [7]. Sucrose in the cytoplasm is converted to fructose and glucose by neutral invertase (NI) and transported to the vacuoles, where it is hydrolyzed to glucose and fructose by the vacuolar invertase (acid invertase, AI). Fructose and glucose are phosphorylated to 6-phosphate fructose (F6P) and 6-phosphate glucose (G6P) with the action of fructokinase (FK) and hexokinase (HK), and then participate in the Embden–Meyerhof–Parnas (EMP) and the tricarboxylic acid (TCA) cycles [6]. As one of the key sugar metabolites of pears, sorbitol is linked to the sweetness of fruits, as well as resistance to abiotic stress. Sorbitol can be converted into fructose and glucose through the catalysis of sorbitol dehydrogenase (NAD-SDH and NADP-SDH) and sorbitol oxidase (SOX) [8]. Moreover, sugar metabolism in postharvest fruits is affected by various external factors. For example, the sugar content and enzyme activity of sugarcane stems can be significantly influenced by temperature variation [9], and sugar composition would change in the hypoxia conditions [10].

As a conventional technology in the preservation of fruits and vegetables, controlled atmosphere (CA) storage with different concentrations of CO_2_ is widely used for fresh-cut fruits and vegetables. It has been demonstrated that the controlled atmosphere could retard respiration rate and biologic activity [11], delay senescence [12], and inhibit the growth of microorganisms [13] by adjusting the ratio of O_2_ and CO_2_ to maintain quality and extend the shelf life of fresh-cut products. Studies have shown that high concentrations of CO_2_ can effectively prolong the commercial life and retain the good quality of fruits and vegetables, including mango [14], avocado [15], pineapple [16], cherry [17], guava [18], and strawberry [19]. Our previous work has also revealed that high concentrations of CO_2_ have a positive effect on maintaining color and extending the shelf life of strawberry [20,21] and mandarin fruit [22]. In addition, it is reported that low concentrations of CO_2_ can induce the accumulation of sucrose, fructose, glucose, and sorbitol [23]. Zhu et al. [6] found that CO_2_ increased the activity of SPS, SS, and FK, while decreased the activity of invertase activities (AI and NI), which retains high levels of sucrose, fructose and glucose in apple fruit.

Presently, the studies on sugar metabolism have concentrated on pre-harvest fruits during growth and development, including tomato [24], peach [25], and apple [26], while few studies were carried out on sugar metabolism in postharvest fruits, especially fresh-cut fruits. As a common gas of controlled atmosphere, CO_2_ is widely used in fruit preservation [11], and there are also few studies on the effect of high concentrations of CO_2_ on sugar metabolism of fresh-cut pears. Therefore, our study is aimed to explore the effects of high CO_2_ on fruit quality, soluble sugar content, enzyme activity, and expression levels of key genes related to their metabolism in fresh-cut pear. Our study not only can illuminate why high CO_2_ maintains the high content of soluble sugars in fresh-cut pear, but also reveals the crucial role of key genes and enzymes in regulating mutual conversion of sugars during storage.

## 2. Results

### 2.1. Firmness and Total Soluble Solids (TSS)

**Figure 1:**
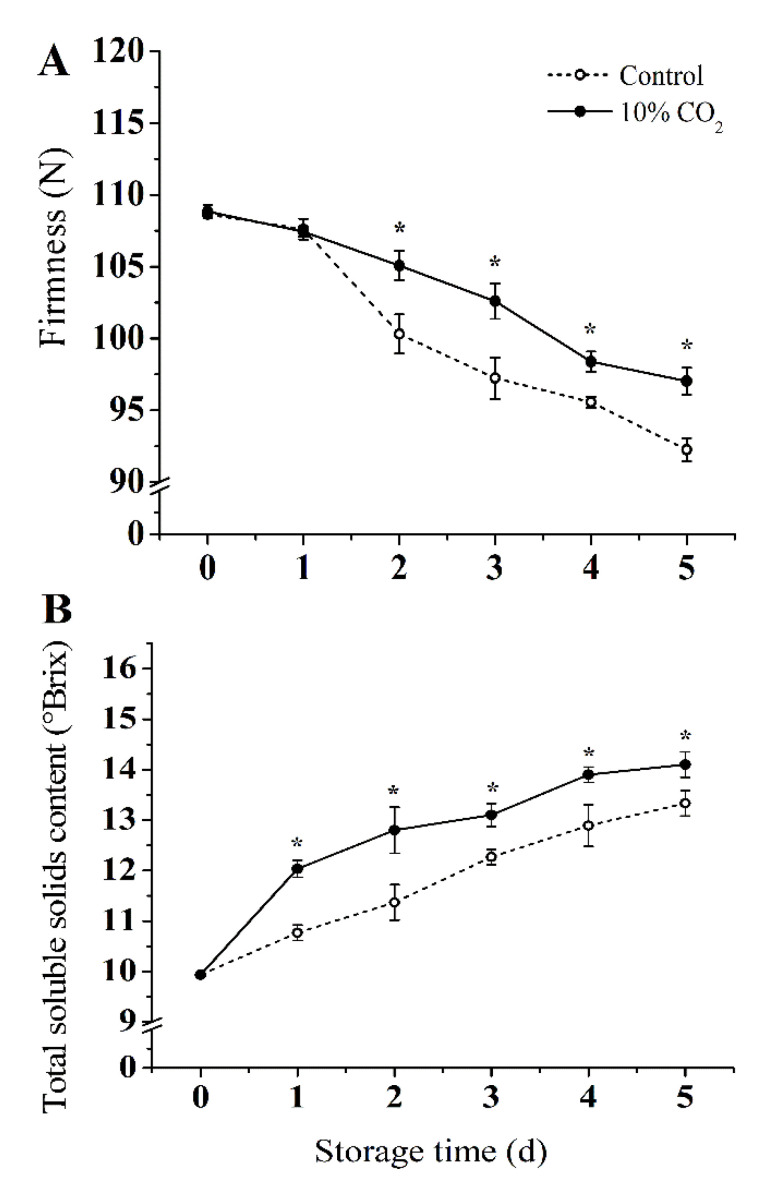
Effect of CO_2_ on firmness (**A**), and total soluble solids (**B**) of fresh-cut pear fruit during storage. Values are presented as means ± SD (*n* = 9). Asterisks (*) indicate significant differences at the same time between controls and CO_2_-treated fruit according to the Student’s-test (*p* < 0.05).

As shown in Figure 1A, the firmness decreased in both CO_2_-treated and control groups during storage. Compared with the control group, 10% CO_2_ treatment delayed the decrease of fruit firmness significantly (*p* < 0.05). At the end of storage, the firmness of the 10% CO_2_-treated group was 98 newton (N) compared to 91 N of the control group, which represents 7.1% higher firmness compared with the control group. As shown in Figure 1B, the 10% CO_2_ treatment accelerated the accumulation of TSS. The TSS content of the 10% CO_2_-treated group was 7.6% higher than the control at the end of storage.

### 2.2. Sucrose, Glucose, Fructose, Sorbitol, and Starch Content

**Figure 2:**
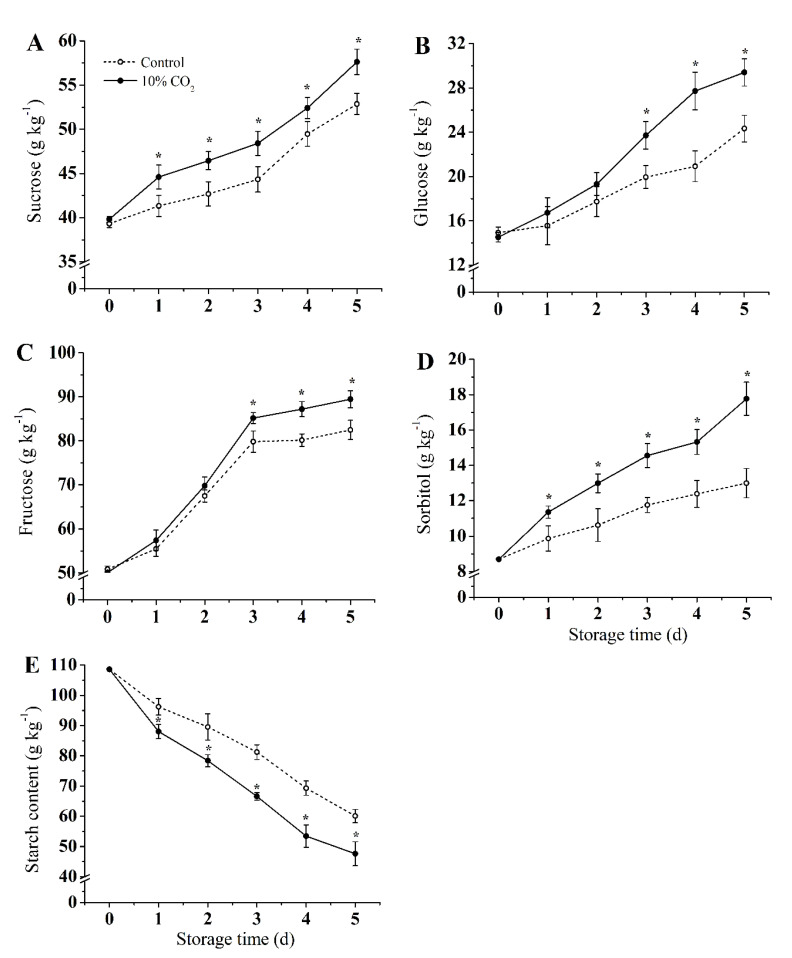
Effect of CO_2_ on sucrose (**A**), glucose (**B**), fructose (**C**), sorbitol (**D**), and starch (**E**) content of fresh-cut pear fruit during storage. Values are presented as means ± SD (*n* = 9). Asterisks (*) indicate significant differences at the same time between controls and CO_2_-treated fruit according to the Student’s-test (*p* < 0.05).

Figure 2 shows the content changes of sucrose, glucose, fructose, sorbitol, and starch. The contents of sucrose, glucose, and fructose gradually increased in both CO_2_-treated and control groups during storage, while the starch content continued to decrease. Compared with the control group, CO_2_ treatment significantly induced the accumulation of sucrose, glucose, fructose, and sorbitol, while the decomposition of starch was promoted (*p* < 0.05). Compared with the control group, sucrose, glucose, fructose, and sorbitol in the CO_2_ treatment group increased by 13.9%, 12.58%, 13.86%, and 24.7%, respectively, and the starch content of the CO_2_ treatment group was reduced by 22% on the fifth day of storage.

### 2.3. SS-Synthesis, SS-Cleavage, AI, NI, Amylase, SPS, HK, and FK Activities

**Figure 3:**
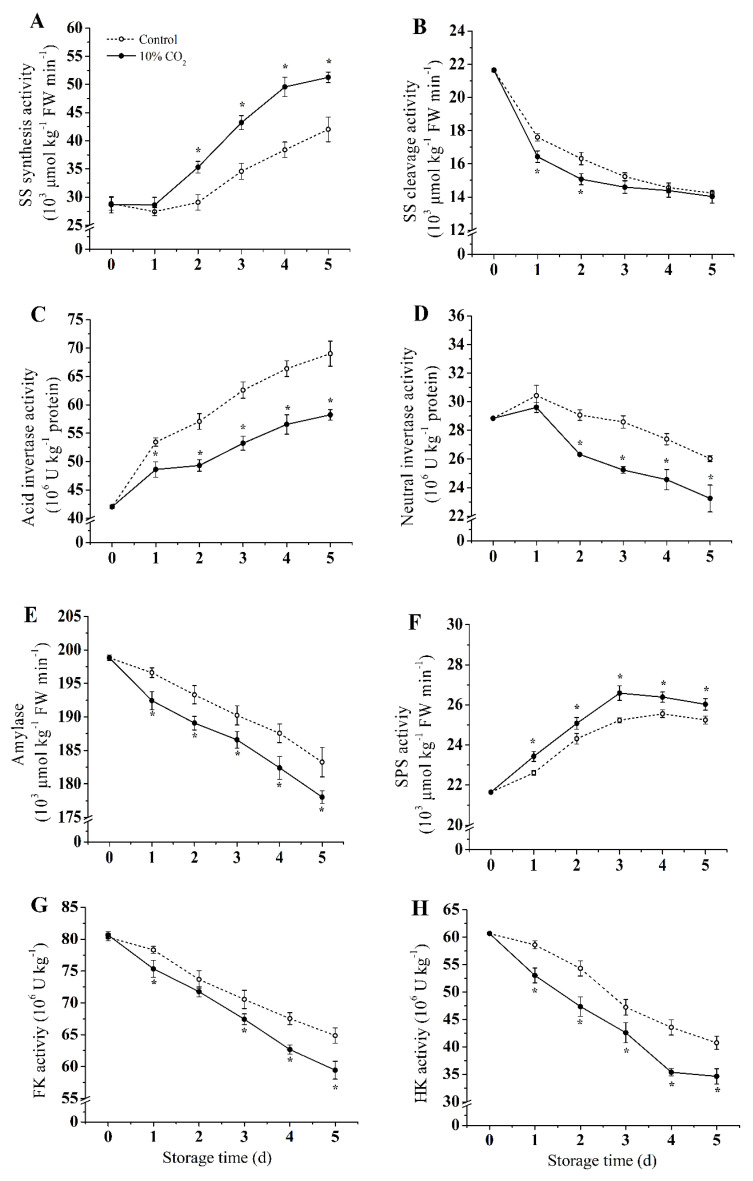
Effect of CO_2_ on activities of sucrose synthase (SS)-synthesis (**A**), SS-cleavage (**B**), acid invertase (AI) (**C**), neutral invertase (NI) (**D**), Amylase (**E**), sucrose phosphate synthase (SPS) (**F**), hexokinase (HK) (**G**), and fructokinase (FK) (**H**) of fresh-cut pear fruit during storage. Values are presented as means ± SD (*n* = 9). Asterisks (*) indicate significant differences at the same time between controls and CO_2_-treated fruit according to the Student’s-test (*p* < 0.05).

Compared with the control group, CO_2_ treatment induced SS-synthesis and significantly increased SPS activity during storage (*p* < 0.05), while SS-cleavage activity was inhibited (Figure 3A,B). At the end of storage, the SS-synthesis activity of the CO_2_-treated group increased by 21%, while SS-cleavage activity was reduced by 21% compared with the control group. During storage, CO_2_ induced SPS activity which peaked on the third day when SPS activity increased by 21%.

The activity of NI, Amylase, FK, and HK continued to decrease, whereas the activity of AI kept increasing during the storage (Figure 3C–E,G,H). Compared with the control group, AI, NI, FK, HK, and amylase activities were inhibited by CO_2_ treatment. In the CO_2_-treated group, the activities of AI, NI, Amylase, FK, and HK were reduced by 26%, 23%, 2.8%, 26% and 21%, respectively, on the fifth day of storage.

### 2.4. SOX, NAD-SDH, and NADP-SDH Activities

**Figure 4:**
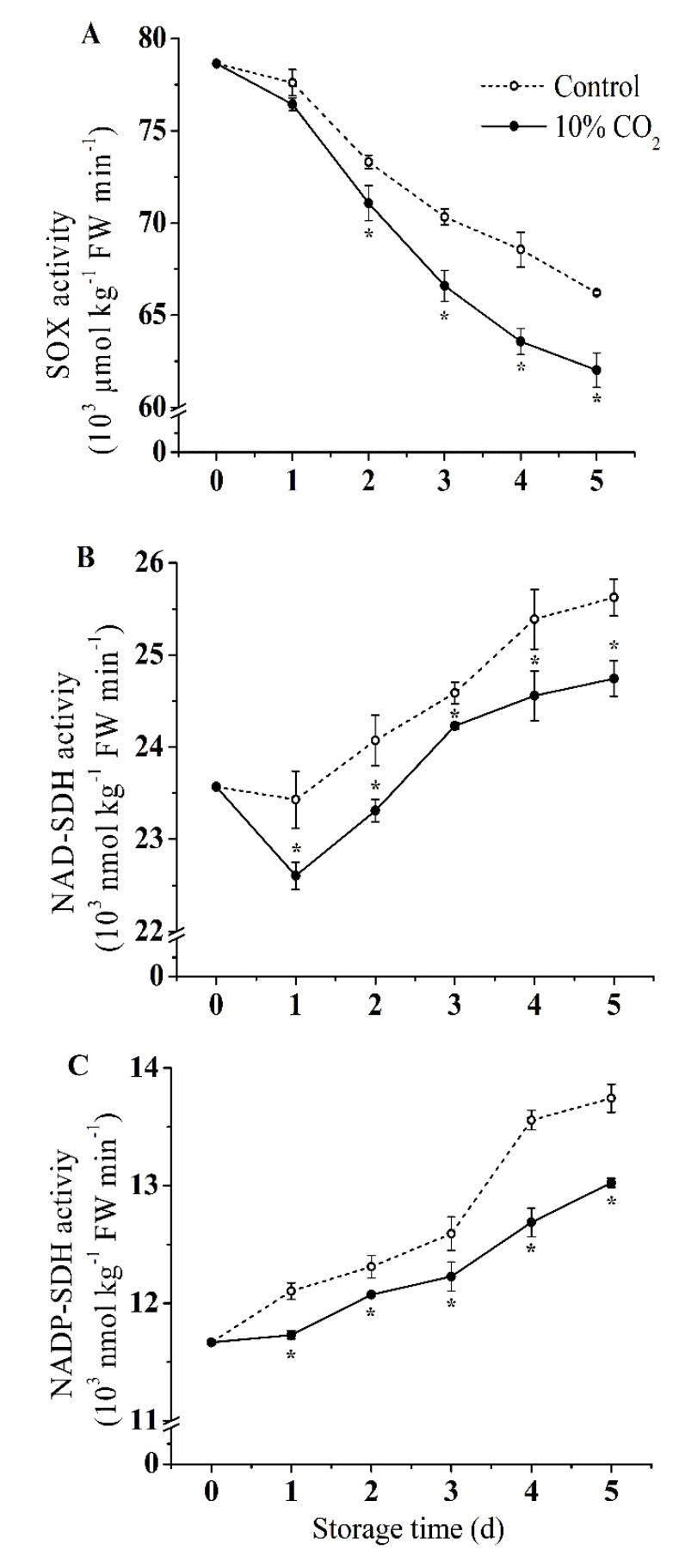
Effect of CO_2_ on activities of SOX (**A**), NAD-SDH (**B**), and NADP-SDH (**C**) of fresh-cut pear fruit during storage. Values are presented as means ± SD (*n* = 9). Asterisks (*) indicate significant differences at the same time between controls and CO_2_-treated fruit according to the Student’s-test (*p* < 0.05).

As shown in Figure 4, the activity of SOX continued to decrease, and NAD-SDH and NADP-SDH continued to increase during storage. CO_2_ treatment significantly inhibited SOX, NAD-SDH and NADP-SDH activities (*p* < 0.05). Compared with the control group, the SOX, NAD-SDH, and NADP-SDH activities of the CO_2_-treated group significantly decreased by 21%, 18% and 22%, respectively, at the end of storage (*p* < 0.05).

### 2.5. Gene Expression of SS-Synthesis, AI, NI, Amylase, SPS, HK, and FK

**Figure 5:**
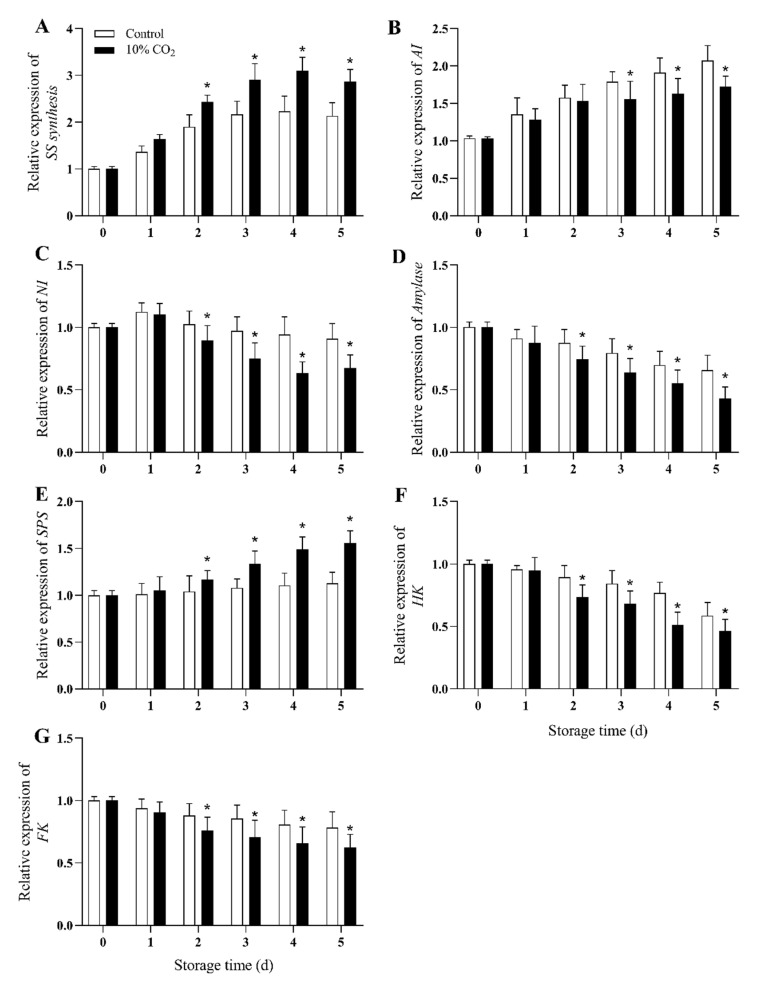
Effect of CO_2_ on gene expression levels of SS-synthesis (**A**), AI (**B**), NI (**C**), Amylase (**D**), SPS (**E**), HK (**F**), and FK (**G**) of fresh-cut pear fruit during storage. Values are presented as means ± SD (*n* = 9). Asterisks (*) indicate significant differences at the same time between controls and CO_2_-treated fruit according to the Student’s-test (*p* < 0.05).

The qRT-PCR results showed that gene expression of SS-synthesis and SPS increased with storage time, and high-concentration CO_2_ treatment significantly increased gene expression of SS-synthesis and SPS during storage (*p* < 0.05) (Figure 5A,E). For CO_2_-treated fruit, SS-synthesis genes (*PpSS-synthesis*) rose to a peak on the fourth day of storage, and declined afterward (Figure 5A). On the fifth day, the expression level of *PpSS-synthesis* and SPS genes (*PpSPS*) decreased by 25.8% and 27.6%, respectively, compared with the control group.

The expression of NI genes (*PpNI*), Amylase genes (*PpAmylase*), HK genes (*PpHK*), and FK genes (*PpFK*) reached a low in the later stages (Figure 5C,D,F,G). The gene expression levels of AI, NI, Amylase, HK, and FK were significantly downregulated in the 10% CO_2_-treated group compared with the control (*p* < 0.05). At the end of storage, the expression of *PpAI, PpNI, PpAmylase, PpFK*, and *PpHK* decreased by 19%, 27%, 34.8%, 21% and 22%, respectively, compared with the control group.

### 2.6. Gene Expression of NAD-SDH and NADP-SDH

**Figure 6:**
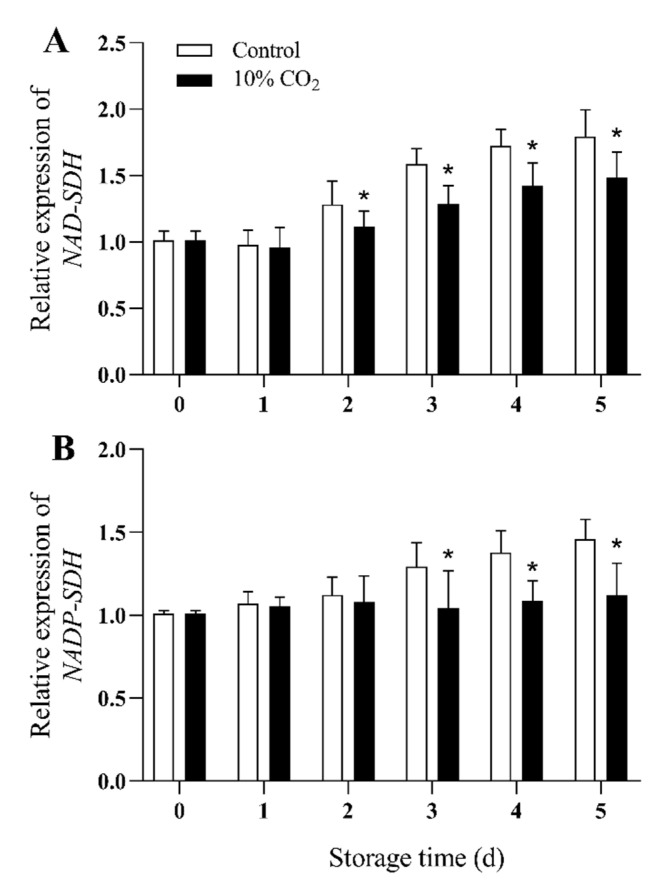
Effect of CO_2_ on gene expression levels of NAD-SDH (**A**), and NADP-SDH (**B**) of fresh-cut pear fruit during storage. Values are presented as means ± SD (*n* = 9). Asterisks (*) indicate significant differences at the same time between controls and CO_2_-treated fruit according to the Student’s-test (*p* < 0.05).

Expression of NAD-SDH genes (*PpNAD-SDH*) and NADP-SDH genes (*PpNADP-SDH*) in fresh-cut pears increased with time in the control group during storage (Figure 6A,B). The expression levels of *PpNAD-SDH* and *PpNADP-SDH* in CO_2_ treated pear fruit were significantly lower than those of the control group during storage (*p* < 0.05), and the *PpNAD-SDH* remained constant in CO_2_-treated fruit during the whole storage. Compared with the control group, the expression levels of *PpNAD-SDH* and *PpNADP-SDH* of the CO_2_-treated group decreased by 18% and 25%, respectively, at the end of storage.

## 3. Discussion

Sugar content is one of the important parameters responsible for the quality of fruits. The effect of its metabolism on the formation of fruit quality cannot be ignored. In postharvest fruits, the changes in sugar composition and content in the fruit are mainly affected by respiration [6]. In addition, sugars are the major osmolytes that accumulate in fruits in response to abiotic stresses [27]. Therefore, sugar metabolism in fruits is mainly affected by surroundings gas composition and abiotic stress [6].

As a typical gas, CO_2_ affects various physiological processes of plants, such as growth, photosynthesis, chlorophyll synthesis, and metabolism, and can reduce the adverse effects caused by abiotic stress [28]. At the postharvest stage, elevated CO_2_ is conventionally utilized to extend fruit shelf life [13,20,26]. Furthermore, CO_2_ has also shown a remarkable effect in retarding browning, inhibiting the growth of microorganisms [13], and delaying softening of fruits and vegetables [20,29]. CO_2_ also affects sugar metabolism and product quality by altering the respiration rate of fruits [30].

Previous studies have shown that CO_2_ contributes to maintain a high level of soluble sugars in apple [6], broccoli, and asparagus [31]. Although various studies have reported that CO_2_ treatment could affect sugar metabolism and quality during the developmental period or integrity of fruits [6]; however, research on the effect of high CO_2_ on sugar metabolism and quality of fresh-cut fruits is lacking. Thus, in our study, the regulation of fruit quality and sugar metabolism using high CO_2_ concentrations in fresh-cut pear were investigated. Obviously, as shown in Figure 1, high levels (10%) of CO_2_ inhibited the decline of firmness and increased TSS content, which was also found in cherry [32], guava [31], apple [33], and strawberry [20] under treatment with elevated CO_2_ concentrations. The results suggested that CO_2_ could regulate the mutual conversion of different sugars and maintain the quality in fresh-cut pear fruit [6,34].

Generally, CO_2_ has been proven to regulate the conversion of soluble sugar in postharvest fruit, e.g., promotes the decomposition of starch and the accumulation of other soluble sugars, which is beneficial it to maintain a high level of sugar content in postharvest fruit [6,34]. However, the starch content of the CO_2_ treatment group decreased significantly compared to the control group, which could be due to the regulation of CO_2_ treatment of sucrose metabolism, promoting the conversion of starch to sucrose. Similarly, Marian et al. [34] found a dramatic loss of starch from broccoli tissues at the beginning of storage under 10% CO_2_ treatment, while the glucose content remained constant, which may be attributed to the conversion of sugars. Regulation of sucrose metabolism is an important mechanism to prevent the quality decline in postharvest fruits [35]. In particular, sucrose is converted into fructose and glucose with the help of invertase [7]. In the present study, CO_2_ treatment inhibited the activities (Figure 3C,D) and the expression levels (Figure 5B,C) of AI and NI. The results indicated that CO_2_ also regulated the conversion and utilization of sucrose in vacuoles, and promoted the conversion of sucrose to a hexose. Wang et al. [2] suggested that the degradation of sucrose under hypoxic conditions may promote the invertase pathway. Zhu et al. [6] found that CO_2_ inhibited activities of AI and NI, and delayed the hydrolysis of sucrose, which lead to a higher level of sucrose in apple fruits. Similar results were also reported for asparagus and broccoli [34].

SS (including SS-cleavage and SS-synthesis) can catalyze the conversion of sucrose into fructose, glucose, and UDPG reversibly [5]. The crucial role of SS is sucrose synthesis [2]. SPS is a critical enzyme in an irreversible reaction, which can convert UDPG and 6-phosphate-fructose to sucrose. The present research indicates that CO_2_ treatment enhanced expressions and activities of SS-synthesis and SPS, and inhibited gene expression and activity of SS-cleavage activity. Previous studies have shown that the transcription level and activity of SS-synthesis were induced under hypoxia conditions [2]; a similar result was also found in apple fruits [6] and peach fruits [10]. Choudhuryet et al. [36] found that the expression level of SPS in banana fruits is regulated by abiotic factors and plant hormones (such as white light and ethylene). Moreover, Yu et al. [37] found that 1-MCP treatment could delay the softening and enhance chilling resistance by inducing SS-synthesis activity and inhibiting the activities of the enzymes related to sucrose degradation. Our results showed that higher enzyme activity in sucrose synthesis and lower enzyme activity in sucrose degradation contribute to maintaining the quality of fresh-cut pear fruits.

HK and FK catalyze the conversion of glucose and fructose to glucose 6-phosphate and fructose 6-phosphate, respectively. Glucose 6-phosphate and fructose 6-phosphate further participate in EMP and other metabolic pathways [7]. In this study, it was found that CO_2_ treatment inhibited HK and FK activities, and gene expression in fresh-cut pear fruits, which caused high levels of glucose and fructose in fruits compared to the control group. In agreement with current results, CO_2_ treatment in apples also showed lower HK and FK activities and higher contents of fructose and glucose [6]. In addition, HK and FK have also been shown to be promoted in tomato root under hypoxic conditions [38], whereas the activities of HK and FK decreased in rice [39], which may be due to the differences in species. Marian et al. [34] reported that HK and FK enzyme activities failed to show significant differences in both CO_2_-treated and control group of asparagus, in disagreement with the present research and was probably due to the difference of tissue structure and the treatment.

As one of the main soluble sugars in pear fruit, sorbitol not only affects sugar metabolism, but also contributes to quality of fruits [40]. Moreover, sorbitol metabolism is affected by environmental conditions. Sorbitol accumulated and involved in regulating osmotic pressure under osmotic stress [8]. Among the Rosaceae fruits, sorbitol was mainly synthesized by photosynthesis during development, and the metabolism of sorbitol in postharvest fruits is primarily based on the mutual transformation with other sugars [40,41]. NAD-SDH and NADP-SDH, SOX, catalyze the conversion of sorbitol to fructose and glucose, respectively [40,41]. Our results showed that CO_2_ treatment induced the accumulation of sorbitol in fresh-cut pear fruits, which may result through the induction of activities and gene expression of NAD^+^-SDH, NADP^+^-SDH, which in turn promote the conversion of fructose and glucose to sorbitol. Moreover, SOX activity was inhibited by CO_2_, indicating that the decomposition of sorbitol was suppressed, and the accumulation of sorbitol was accelerated, which contributed to the enhancement of stress resistance of fresh-cut pear fruits. The above results were consistent with the changes in sorbitol levels, activity and gene expression levels of SOX, NAD-SDH, NADP-SDH under high temperature in pear leaves [40], revealing the significant role of sorbitol in response to stresses.

## 4. Materials and Methods

### 4.1. Pear Fruit, Treatment, and Storage

‘Cuiguan’ pears (*Pyrus pyrifolia* Nakai cv. Cuiguan) were collected from a local market in Zhejiang, China, and were transported to the laboratory within 2 h. Pears with uniform size and no blemishes were selected and washed with potable water. Pears were cut in half (lengthwise) and peeled and cored. Eventually, 864 pear fruit were selected and divided randomly into 4 groups. For each group, every two pear fruit were put on one plastic container. Samples were packaged in a hermetically sealed polypropylene plastic container (22 × 13.5 × 4 cm), with two inlet ports, filled with 10 (*v*/*v*) 10% CO_2_ (with 79% N_2_ and 11% O_2_, respectively) for treatment, and the gas concentrations were kept constant during storage. A container flow-through gas system was used to regulating the gas composition of the container and a portable gas analyzer (MOCON Europe A/S, Ringsted, Denmark) was used to determine atmosphere. All samples were stored at 5 °C for 5 d. Air (21% O_2_ and 79% N_2_) was used under the same situation as a control treatment. The quality indices and physiological parameters were determined daily. There were twelve containers for each treatment, and each treatment was conducted independently for nine biological replications.

### 4.2. Determination of Firmness and Total Soluble Solids (TSS)

Firmness was measured by using a texture analyzer (TAXT2i, Stable Micro System, Godalming, Surrey, UK) equipped with a cylindrical probe (5 mm diameter) and the test speed was 1.5 mm/s. The maximum force was recorded and expressed in Newton (N).

TSS was determined with a refractometric saccharometer (Atago, Minato-ku, Tokyo, Japan). The data were expressed in Brix. 27 July 2020.

### 4.3. Determination of Sucrose, Glucose, Fructose, and Sorbitol Content

The sucrose, glucose, fructose, sorbitol contents were determined via high performance liquid chromatography (HPLC) method as described previously [4]. The 2 g frozen sample was ground in 5 mL 80% ethanol solution and then incubated at 80 °C for 1 h. The homogenate was centrifuged at 10,000× *g* for 20 min, and the supernatant was diluted 20 times with deionized water and passed through a 0.22 μm membrane filter. The sucrose, glucose, fructose, sorbitol contents were determined by HPLC (Shimadzu DGU-20A, Kyoto, Japan) equipped with a 5 μm XB-NH2 column (4.6 × 250 mm, Welch Materials, Inc., Shanghai, China); the injection volume was 20 μL. Acetonitrile-water (75:25, *v*/*v*) was used as the mobile phase with a flow rate of 1.0 mL min^−1^. The sucrose, glucose, fructose, and sorbitol contents were determined according to the retention time (min) of the standard compounds. The results were expressed based on fresh weight (FW) as g kg^−1^.

### 4.4. Determination of Starch Content

The starch content was determined by the method of Duan et al. [42] with slight modifications. Frozen sample (1 g) was ground with 5 mL of methanol, chloroform and water (12:5:2, *v*/*v*/*v*) and centrifuged at 7000× *g* for 10 min. The supernatant was discarded and the precipitate was dissolved in 15 mL of 50% (*v*/*v*) methanol, then mixed with 5 mL of dimethyl sulfoxide and 1.25 mL of 8 M HCl, and incubate at 60 °C for 75 min. After adjusting the pH of the sample to 4.5–5.0, the volume was adjusted to 20 mL with water and the absorbance measured at 620 nm by colorimetry. The results were expressed based on FW as g kg^−1^.

### 4.5. Determination of SS-Synthesis, SS-Cleavage, AI, and NI Activities

AI, SS-synthesis, and SS-cleavage were extracted and determined by the method of Duan et al. [42] with slight modifications. Frozen sample (4.0 g) was ground with 10.0 mL of 100 mM sodium phosphate buffer (pH 7.5, containing 10.0 mM MgCl_2_, 1.0 mL/L β-mercaptoethanol, 1.0 g/L PVPP) and mixed with 1.0 mL/L Triton X100. The mixture was centrifuged at 12,000× *g* for 15 min at 4 °C, and the supernatant was collected for testing. The mixture containing 2 mL of AI extract and 1 mL of the reaction solution (pH 5, containing 200 mM sodium acetate, 20 mM ethylenediaminetetraacetic acid (EDTA), 250 mM sucrose) were incubated at 30 °C for 30 min, then 1 mL of 3,5-dinitrosalicylic acid (DNS) was added to stop the reaction. After keeping in boiling water for 10 min, the absorbance was measured at 540 nm. The glucose (1.0 mg mL^−1^) standard curve was used to calculate AI activity. The NI activity was measured by a NI activity detection kit (Shanghai Solarbio Biotechnology Co., Ltd., Shanghai, China) according to the product instructions. AI and NI activities were expressed as U kg^−1^ protein.

For SS-synthesis activity determination, the mixture (70 μL) of 4.0 mM UDPG, 100 mM 2-[4-(2-hydroxyethyl) piperazin-1-yl] ethanesulfonic acid (HEPES)-NaOH (pH 8.0) buffer, 15 mM MgCl_2_, 0.06 M fructose and 40 μL enzyme extract was for reaction, and 5.0 mM NaOH was added to the mixture to stop the reaction. After keeping in boiling water for 5 min, the mixture was incubated for 10 min with 80% anthrone sulfate, and the absorbance was measured at 620 nm. SS-synthesis activity was expressed based on FW as μmol kg^−1^ min^−1^.

For SS-cleavage activity determination, the 0.4 mL of enzyme extract and 0.5 mL of HEPES-NaOH buffer solution (pH 5.5, containing 50 mM UDP, 50 mM sucrose, 100 mM NaF) were mixed and incubated at 30 °C for 30 min, 1 mL of DNS was added in the mixture to stop the reaction. After keeping in boiling water for 10 min, the absorbance was measured at 520 nm. The glucose (1.0 mg mL^−1^) standard curve was used to calculate enzyme activity, and SS-cleavage activity was expressed based on FW as μmol kg^−1^ min^−1^.

### 4.6. Determination of Amylase and SPS Activities

The amylase and SPS activities were determined according to the method of Duan et al. [42] with slight modifications. Frozen sample (1 g) was homogenized with 5 mL 100 mM potassium phosphate buffer (pH 7.5; containing 2.5 mM dithiothreitol, 2% PVP, 5 mM MgCl_2_ and 0.1% Triton X) and centrifuged at 10,000× *g* for 20 min at 4 °C. The precipitate was collected and dissolved in 5 mL of 20 mM potassium phosphate buffer (pH 7.5; containing 25 mM MgCl_2_ and 2.5 mM dithiothreitol) for testing.

For amylase activity determination, the mixture of 0.2 mL enzyme extract and 0.3 mL of 3.5% tris (hydroxymethyl) aminomethane (Tris) (*w*/*v*) were mixed with 2 mL of 1% starch solution, and incubated at 37 °C for 10 min (the control group was boiled for 10 min), the 4 mL 0.1 M NaOH was added to stop the reaction. The absorbance was measured at 590 nm. The maltose (1.0 mg mL^−1^) standard curve was used to calculate enzyme activity, and amylase activity was expressed based on FW as μmol kg^−1^ min^−1^.

For SPS determination, the mixed reaction system contained 100 mM HEPES-NaOH buffer (pH 8.0), 10 mM uridine diphosphate glucose, 5 mM fructose-6-Phosphate, 15 mM glucose 6-phosphate, 15 mM MgCl_2_, and enzyme extract. The mixture was incubated at 37 °C for 30 min and boiled for 10 min, the 5 mM NaOH was added in the mixture to stop the reaction. After cooling for 10 min, the absorbance was measured at 490 nm. The sucrose (1.0 mg mL^−1^) standard curve was used to calculate enzyme activity, and SPS activity was expressed based on FW as μmol kg^−1^ min^−1^.

### 4.7. Determination of FK and HK Activities

FK activity was measured according to the method of Zhu et al. [6] and with slight modifications. Frozen samples (2 g) were homogenized with 3 mL of 50 mM HEPES-NaOH buffer (pH 7.0) (containing 2.5 mM dithiothreitol (DTT), 1 mM EDTA, 10% glycerol, 0.05% Triton-X 100 and 0.5 mM bovine serum albumin (BSA)) and centrifuged at 12,000× *g* for 20 min, the supernatant was collected for testing. The reaction system included 1 mL of enzyme extract, 50 mM HEPES-NaOH (pH 7.0), 1 mM ATP, 1 mM KCl, 1 mM NAD, and 1 U glucose-6-phosphate dehydrogenase (G-6-PDH). The 1 U phosphoglucose isomerase (PGI) and 2 mM fructose were added to the mixture to start the reaction. The absorbance was measured at 340 nm, and SPS activity was expressed as U kg^−1^ protein. The HK activity was measured by using a HK activity detection kit (Shanghai Solarbio Biotechnology Co., Ltd., Shanghai, China) according to the instructions. HK activity was expressed as U kg^−1^ protein.

### 4.8. Determination of SOX, NAD-SDH, and NADP-SDH Activities

The SOX activity was determined according to the method of Wang et al. [8] with slight modifications. Frozen sample (0.7 g) was ground with 3.5 mL of 50 mM HEPES-NaOH buffer (pH 7.5) (contains 2.5 mM dithiothreitol, 5 mM MgCl_2_, 0.05% TritonX-100, 1 mM EDTA, 0.1% BSA, and 2% polyvinyl pyrrolidone (PVP)) and centrifuged at 10,000× *g* at 4 °C for 15 min, and the supernatant was collected for testing. The SOX reaction system contained 100 mM citrate-trisodium citrate buffer (pH 4.0), 200 mM sorbitol, and 1.5 mL of enzyme extract. The mixtures were incubated at 30 °C for 30 min, and 0.5 mL of 3,5-dinitrosalicylic acid (DNS) was added to stop the reaction. After boiling for 5 min, the mixture was centrifuged at 4000× *g* for 10 min. The absorbance value was measured at 540 nm. The glucose (1.0 mg mL^−1^) standard curve was used to calculate enzyme activity and SOX activity was expressed based on FW as μmol kg^−1^ min^−1^.

The NAD-SDH and NADP-SDH activities were determined according to the method of Wang et al. [8] with slight modifications. Frozen samples (3 g) were homogenized with 10 mL 200 mM potassium phosphate buffer (pH 7.0) (contained 10 mM ascorbic acid, 20 mM 2-mercaptoethanol, 0.1% Triton-X 100 and 10% PVPP) and centrifuged at 12,000× *g* for 20 min, and the supernatant was collected for testing. The reaction system of NAD-SDH included 30 mM Tris-HCl buffer (pH 9.6), 1 mM NAD^+^, and 275 mM sorbitol. The reaction system of NADP-SDH included 30 mM Tris-HCl buffer (pH 8.5), 1 mM NADP^+^, 3 mM MgCl_2_ and 275 mM sorbitol. The absorbance was measured at 340 nm. NAD-SDH and NADP-SDH activities expressed based on FW as nmol kg^−1^ min^−1^, and protein content was measured by the Coomassie brilliant blue method [43].

### 4.9. Determination of Related Gene Expression by Real-Time Quantitative PCR (qRT-PCR)

Total RNA was extracted using a QIAGEN RNeasy Mini kit (QIAGEN GmbH-Hilden, Dusseldorf, Germany). The purity and concentration of total RNA were measured at 260 nm by using a spectrophotometer (NanoDropND-2000, Thermo, Waltham, MA, USA). The Prime Script RT Reagent Kit (Takara Biomedical, Kyoto, Japan) was used to synthesize the first-strand cDNA.

The sequence of gene primers was designed using OMIGA 2.0 software, as shown in Table 1. The total volume of the qRT-PCR reaction was 20.0 μL, including 10.0 μL SYBR Green PCR Premix Ex Taq ™ (Takara Biomedical, Kyoto, Japan), 0.5 μL ROX reference dye II, 0.5 μL forward and reverse primers (10 μ mol L^−1^), 5 ng cDNA and 6.0 μL ultrapure water. The reaction was carried out on an ABI 7500 instrument (Applied Biosystems, Foster City, CA, USA). The operational conditions were as follows: at 95 °C for 10 s, at 95 °C for 5 s with 40 cycles, and at 60 °C for 34 s. The amplification efficiency and primer specificity were confirmed by comparing the slope of the amplification curve. Pear actin gene (*PpActin*, JN684184) was used as an internal reference gene to quantify the amounts of qRT-PCR products of different genes. The 2^−ΔΔCT^ method was used to calculate the result.

### 4.10. Statistical Analysis

The data were statistically treated by using one-way variance analysis (ANOVA) and Student’s t-test using SPSS 24.0 (SPSS Inc., Chicago, IL, USA), and a *p*-value < 0.05 was considered significant.

## 5. Conclusions

In conclusion, this study revealed that CO_2_ treatment induced activity and gene expression levels of SS-synthesis and SPS, while activity and gene expression levels of amylase, AI, NI, SS-cleavage, FK, HK, SOX, NAD-SDH, and NADP-SDH were inhibited. The decrease in firmness and total soluble solids were delayed by CO_2_ treatment. In addition, contents of glucose, fructose, sucrose, and sorbitol increased; meanwhile, the levels of starch decreased during storage. The above results suggested that CO_2_ treatment maintained a higher level of soluble sugars by regulating the genes encoding enzymes related to sugar metabolism, which benefits against wound stress and maintains a higher quality of fresh-cut pear fruit.

Our results indicated that CO_2_ treatment is favorable for the metabolism of soluble sugars and for the quality maintenance of fresh-cut pear fruit during storage. However, the fresh-cut pear fruit is different due to the short shelf life caused by increased respiration rate. Further studies should be conducted to retard anaerobic respiration to extend the shelf life of fresh-cut pear fruit.

## Figures and Tables

**Table 1:** Primer sequences used for real-time quantitative PCR (qRT-PCR).

Gene	Forward Primer (5′–3′)	Reverse Primer (5′–3′)	Product Size (bp)
Actin	TCCCACATGCCATCCTTCGTTTG	GTCCCTCACAATTTCCCGCTCAG	122
SPS	GGCGGGAAATGACTGGGTGAAC	CGGAGGAGCAGCGATGATTTGG	90
SS-synthesis	AGCAGCACCAACCTTAGCAATCC	CGGAGGAGCAGCGATGATTTGG	119
NI	CAAGCACGCCTGTTCCAGACC	GCAGGAGAAGGCATTCACGAGTTC	129
AI	CGCTACTCCCTGACGGTAACATTG	ATACGCTAGGCATTGCACTTGGAC	80
Amylase	AACCGAACCCAGATCAAGATGCAC	CGAGTCCTTCAGCCTCCAAAGC	146
NAD-SDH	ATGGCTGCTTGGCTCGTTGATG	TCACTTCCGCAAATGCCGACAG	116
NAD-MDH	TCCGTCCCCGAGCGTAAAGTC	ACCAACATCAGCAGCAACTCCAG	147
FK	ACAAACTGAGAGATGCGCTCG	GCAATGCCGGAATAGCACCT	81
HK	TCCTTGAGTTTGCTCCCGAC	TGGAGTGGGGTAACTTTCGC	292

## References

[1] Borsanie J., Budde C.O., Porrini L., Lauxmann M.A., Lombardo V.A., Murray R., Andreo C.S., Drincovich M.F., Lara M.A. (2009). Carbon metabolism of peach fruit after harvest: Changes in enzymes involved in organic acid and sugar level modifications. J. Exp. Bot..

[2] Wang Y.J., Liu L., Wang Y., Tao H.X., Fan J.L., Zhao Z.Y., Guo Y.P. (2019). Effects of soil water stress on fruit yield, quality and their relationship with sugar metabolism in ‘Gala’ apple. Sci. Hortic..

[3] Ruan Y.L., Jin Y., Yang Y.J., Li G.J., Boyer J.S. (2012). Sugar input, metabolism, and signaling mediated by invertase: Roles in development, yield potential, and response to drought and heat. Mol. Plant..

[4] Li D., Zhang X.C., Li L., Aghdam M.S., Luo Z.S. (2019). Effect of exogenous sucrose on anthocyanin synthesis in postharvest strawberry fruit. Food Chem..

[5] Shi K.K., Liu Z.C., Wang J.W., Zhu S.H., Huang D.D. (2019). Nitric oxide modulates sugar metabolism and maintains the quality of red raspberry during storage. Sci. Hortic..

[6] Zhu Z., Liu R.L., Li B.Q., Tian S.P. (2013). Characterisation of genes encoding key enzymes involved in sugar metabolism of apple fruit in controlled atmosphere storage. Food Chem..

[7] Li M.J., Feng F.J., Cheng L.J. (2012). Expression patterns of genes involved in sugar metabolism and accumulation during apple fruit development. PLoS ONE..

[8] Wang Z., Cao J.K., Jiang W.B. (2016). Changes in sugar metabolism caused by exogenous oxalic acid related to chilling tolerance of apricot fruit. Postharvest Biol. Biotechnol..

[9] Mao L.C., Que F., Wang G.Z. (2006). Sugar metabolism and involvement of enzymes in sugarcane (*Saccharum officinarum* L.) stems during storage. Food Chem..

[10] Lara M.V., Budde C.O., Porrini L., Borsani J., Murray R., Andreo C.S., Drincovich M.F. (2011). Peach (*Prunus Persica*) fruit response to anoxia: Reversible ripening delay and biochemical changes. Plant. Cell Physiol..

[11] Li S.F., Zhang L.H., Liu M.P., Wang X.Y., Zhao G.Y., Zong W. (2017). Effect of poly-ε-lysine incorporated into alginate-based edible coatings on microbial and physicochemical properties of fresh-cut kiwifruit. Postharvest Biol. Biotechnol..

[12] Ali S., Khan A.S., Malik A.U., Anjum M.A., Nawaz A., Shah H.M.S. (2019). Modified atmosphere packaging delays enzymatic browning and maintains quality of harvested litchi fruit during low temperature storage. Sci. Hortic..

[13] Caleb O.J., Mahajan P.V., AI-Said F.A., Opara U.L. (2013). Modified Atmosphere Packaging Technology of Fresh and Fresh-cut Produce and the Microbial Consequences—A Review. Food Bioprocess. Technol..

[14] Ramayya N., Niranjan K., Duncan E. (2012). Effects of modified atmosphere packaging on quality of Alphonso mangoes. J. Food Sci. Technol..

[15] Sellamuthu P.S., Mafune M., Sivakumar D., Soundy P. (2013). Thyme oil vapour and modified atmosphere packaging reduce anthracnose incidence and maintain fruit quality in avocado. J. Sci. Food Agric..

[16] Finnegan E., Mahajan P.V., O’Connell M., Francis G.A., Beirne D. (2013). Modelling respiration in fresh-cut pineapple and prediction of gas permeability needs for optimal modified atmosphere packaging. Postharvest Biol. Biotechnol..

[17] Colgecen I., Aday M.S. (2015). The efficacy of the combined use of chlorine dioxide and passive modified atmosphere packaging on sweet cherry quality. Postharvest Biol. Biotechnol..

[18] Antala D.K., Varshney A.K., Davara P.R., Sangani V.P. (2015). Modified atmosphere packaging of guava fruit. Packag. Technol. Sci..

[19] Blanch M., Álvarez I., Sanchez-Ballesta M.T., Escribano M.I., Merodio C. (2019). Involvement of fatty acids in the response to high CO_2_ and low temperature in harvested strawberries. Postharvest Biol. Biotechnol..

[20] Yang M.Y., Ban Z.J., Luo Z.S., Li J.H., Lu H.Y., Li D., Chen C.K., Li L. (2020). Impact of elevated O_2_ and CO_2_ atmospheres on chemical attributes and quality of strawberry (*Fragaria × ananassa* Duch.) during storage. Food Chem..

[21] Li D., Zhang X.C., Qu H.X., Li L., Luo Z.S. (2020). Delaying the biosynthesis of aromatic secondary metabolites in postharvest strawberry fruit exposed to elevated CO_2_ atmosphere. Food Chem..

[22] Lu Y., Li D., Li L., Belwal T., Luo Z.S. (2020). Effects of elevated CO_2_ on pigment metabolism of postharvest mandarin fruit for degreening. Food Chem..

[23] Cao S., Yang Z., Zheng Y. (2013). Sugar metabolism in relation to chilling tolerance of loquat fruit. Food Chem..

[24] Chen J.L., Gilles V., Shaozhong K., Bertin N., Gautier H., Génard M. (2020). Fruit water content as an indication of sugar metabolism improves simulation of carbohydrate accumulation in tomato fruit. J. Exp. Bot..

[25] Li X.W., Liu P., Zhou J.Y., Su M.S., Ye Z.W. (2020). Effects of exogenous application of GA_4+7_ and NAA on sugar accumulation and related gene expression in peach fruits during developing and ripening stages. J. Plant. Growth Regul..

[26] Li M.J., Li P.M., Ma F.W., Dandekar A.M., Cheng L.L. (2018). Sugar metabolism and accumulation in the fruit of transgenic apple trees with decreased sorbitol synthesis. Hortic. Res..

[27] Lin Q., Xie Y.J., Guan W.Q., Duan Y.Q., Wang Z.D., Sun C.D. (2019). Combined transcriptomic and proteomic analysis of cold stress induced sugar accumulation and heat shock proteins expression during postharvest potato tuber storage. Food Chem..

[28] Pandey R., Zinta G., AbdElgawad H., Ahmad A., Jain V., Janssens I.A. (2015). Physiological and molecular alterations in plants exposed to high CO_2_ under phosphorus stress. Biotechnol. Adv..

[29] Cortellino G., Piazza L., Spinelli L., Torricelli A., Rizzolo A. (2016). Influence of maturity degree, modified atmosphere and anti-browning dipping on texture changes kinetics of fresh-cut apples. Postharvest Biol. Biotechnol..

[30] Thewes F.R., Brackmann A., Neuwald D.A. (2019). Dynamics of sugars, anaerobic metabolism enzymes and metabolites in apples stored under dynamic controlled atmosphere. Sci. Hortic..

[31] Teixeira G.H.A., Júnior L.C.C., Ferraudo A.S., Durigan J.F. (2016). Quality of guava (*Psidium guajava* L. cv. Pedro Sato) fruit stored in low-O_2_ controlled atmospheres is negatively affected by increasing levels of CO_2_. Postharvest Biol. Biotechnol..

[32] Davarynejad G.H., Aryanpooya Z., Persely S.Z. (2010). Effect of modified atmosphere packaging on fresh sour cherry fruit quality. Acta Hortic..

[33] Mditshwa A., Fawole O.A., Vries F., Merwe K.V.D., Crouch E., Opara U.L. (2017). Repeated application of dynamic controlled atmospheres reduced superficial scald incidence in ‘Granny Smith’ apples. Sci. Hortic..

[34] Marian J.M., Lindsay A.G., Julian A.H., Paul L.H. (2004). Sugar metabolism and compartmentation in asparagus and broccoli during controlled atmosphere storage. Postharvest Biol. Biotechnol..

[35] Ge Y.H., Wei M.L., Li C.Y., Chen Y.R., Duan B., Li X., Tang Q., Li X.H. (2019). Changes in the sucrose metabolism in apple fruit following postharvest acibenzolar-S-methyl treatment. J. Sci. Food Agric..

[36] Choudhury S.R., Roy S., Das R., Sengupta D.N. (2008). Differential transcriptional regulation of banana sucrose phosphate synthase gene in response to ethylene, auxin, wounding, low temperature and different photoperiods during fruit ripening and functional analysis of banana SPS gene promoter. Planta.

[37] Yu L.N., Shao X.F., Wei Y.Y., Xu F., Wang H.F. (2017). Sucrose degradation is regulated by 1-methycyclopropene treatment and is related to chilling tolerance in two peach cultivars. Postharvest Biol. Biotechnol..

[38] Gharbi I., Ricard B., Rolin D., Maucourt M., Andrieu M., Bizid E., Smiti S., Brouquisse R. (2007). Effect of hexokinase activity on tomato root metabolism during prolonged hypoxia. Plant. Cell Environ..

[39] Mustroph A., Albrecht G. (2003). Tolerance of crop plants to oxygen deficiency stress: Fermentative activity and photosynthetic capacity of entire seedlings under hypoxia and anoxia. Physiol. Plant..

[40] Yu C.Y., Cheng H.Y., Cheng R., Qi K.J., Gu C., Zhang S.L. (2019). Expression analysis of sorbitol transporters in pear tissues reveals that PbSOT6/20 is associated with sorbitol accumulation in pear fruits. Sci. Hortic..

[41] Liu D.F., Ni J.B., Wu R.Y., Teng Y.W. (2013). High temperature alters sorbitol metabolism in *pyrus pyrifolia* leaves and fruit flesh during late stages of fruit enlargement. J. Am. Soc. Hortic. Sci..

[42] Duan B., Ge Y.H., Li C.Y., Gao X.N., Tang Q., Li X., Wei M.L., Chen Y.R. (2019). Effect of exogenous ATP treatment on sucrose metabolism and quality of ‘Nanguo’ pear fruit. Sci. Hortic..

[43] Bradford M.M. (1976). A rapid and sensitive method for the quantitation of microgram quantities of Protein Utilizing the Principle of Protein-Dye Binding. Anal. Biochem..

